# Green synthesis of plant-derived ZnO nanoparticles: Characterization, pharmacokinetics, molecular interactions, and *in-vitro* antimicrobial and antifungal evaluation

**DOI:** 10.17305/bb.2025.12090

**Published:** 2025-05-15

**Authors:** Samira Jebahi, Riadh Badraoui, Ghada Ben Salah, Fadia Ben Taheur, Faten Brahmi, Mohsen Mhadhbi, Talel Bouhamda, Saoussen Jilani, Bandar Aloufi, Mohd Adnan, Arif J Siddiqui, Abdel Moneim E Sulieman, Ines Karmous

**Affiliations:** 1 Biology and Environmental Department, Insitute of Applied Biology of Medenine (ISBAM), University of Gabes, Medenine, Tunisia; 2 Research Laboratory on Energy and Matter for Nuclear Sciences Development (LR16CNSTN02), National Center for Nuclear Sciences and Technologies, Sidi Thabet Technopark 2020 Ariana, Tunisia; 3 Department of Biology, College of Science, University of Ha’il, Ha’il, Saudi Arabia; 4 Section of Histology-Cytology, Medicine Faculty of Tunis, University of Tunis El Manar, La Rabta-Tunis, Tunisia; 5 Department of Pharmacology and Toxicology, College of Pharmacy, Qassim University, Al Qassim, Saudi Arabia; 6 Laboratory of Analysis, Treatment and Valorization of Environmental Pollutants and Products, Faculty of Pharmacy, University of Monastir, Rue Ibn Sina, Monastir, Tunisia; 7 Department of Chemistry, College of Science, University of Ha’il, Ha’il, Saudi Arabia; 8 Laboratory of Useful Materials, National Institute of Research and Physicochemical Analysis, Technopole Sidi Thabet, Ariana, Tunisia; 9 Arid Land Region Institute of Medenine (IRA), Medenine, Tunisia; 10 Plant Toxicology and Molecular Biology of Microorganisms, Faculty of Sciences of Bizerte, Zarzouna, Tunisia

**Keywords:** Antibacterial and antifungal activities, biosynthesis, nanotechnology, phytochemicals, zinc oxide nanoparticles, ZnONPs, computational modeling

## Abstract

Nowadays, nanoparticles (NPs) are used to counteract various medicinal and industrial problems. This study aimed to biosynthesize zinc oxide NPs (ZnONPs) from the plant species *Aloe vera* L., *Peganum harmala* L., *Retama monosperma* L., and *Thymelaea hirsuta* L. The biosynthesized ZnONPs were referred to as “Thymhirs.bio-ZnONP,” “Aloever.bio-ZnONP,” “Retam.bio-ZnONP,” and “Harm.bio-ZnONP.” A UV-visible spectrophotometer, granulometry, Fourier transform infrared spectroscopy, and electron paramagnetic resonance were used for physicochemical characterization. Pharmacokinetics and antimicrobial effects were explored using combined *in vitro* and computational assays. An abundance of phenolic acids and flavonoids was observed, particularly rutin, quinic acid, apigenin-7-O-glucoside, and cirsiliol, which may act as reducing, stabilizing, and capping agents in the biosynthesis. ZnONPs demonstrated strong antimicrobial activity against various bacterial, fungal, and yeast strains, highlighting their potential medicinal applications. This inhibitory activity can be attributed to the effect of the plant-based ZnO nanosized particles more than to the plant extracts or Zn salt. Computational modeling revealed that the identified phytochemicals (phenolic acids and flavonoids) bound Tyrosyl-tRNA Synthetase (TyrRS) from *S. aureus* (1JIJ), aspartic proteinase from *C. albicans* (2QZW), and wheat germ agglutinin (2UVO) with considerable affinities, which, together with molecular interactions and pharmacokinetics, satisfactorily support the *in vitro* antimicrobial findings. This study lays the groundwork for future research and pharmaceutical explorations aimed at harnessing the likely beneficial properties of green-synthesized ZnONPs for medicinal and therapeutic purposes, particularly their antimicrobial effects.

## Introduction

The engineering of nanoparticles (NPs) has garnered significant attention due to their potential applications across various domains in science and technology [1, 2]. NPs are nanomaterials ranging in size from 10 to −100 nm and possess a higher surface area-to-volume ratio compared to bulk materials. Traditional methods for NP synthesis, including chemical and physical approaches, are often time-consuming, costly, and environmentally hazardous. Consequently, green synthesis has emerged as a faster, more cost-effective, and eco-friendly alternative [3]. Moreover, biosynthetic routes often yield NPs with more uniform size and morphology than conventional physicochemical methods [4]. Green synthesis of metal and metal oxide NPs typically involves plant extracts or microorganisms, such as bacteria, fungi, algae, and yeast [5–7]. Several plant species have been employed in the biosynthesis of zinc oxide NPs (ZnONPs), including *Sageretia thea* [8], *Zingiber officinale* [9], *Catharanthus roseus* [10], *Laurus nobilis L.* [11], *Cannabis sativa* [12], *Ceratonia siliqua* [13], and *Artemisia vulgaris* [14]. ZnONPs have been widely studied for medicinal applications, including drug delivery, antimicrobial and antioxidant activities, and disease diagnostics [15, 16]. Biologically synthesized NPs have also demonstrated effectiveness as antimicrobial agents, drug carriers, and components in medical materials [17, 18]. To the best of our knowledge, this is the first study focusing on the green synthesis of ZnONPs using *Thymelaea hirsuta L.*, *Aloe vera L*., *Retama monosperma L*., and *Peganum harmala L.*—plants known for their medicinal properties and ability to thrive in arid desert environments. *Thymelaea hirsuta L.* (commonly known as Mitnan) is a perennial, evergreen desert shrub belonging to the Thymelaeaceae family, growing up to 2 m tall. It is native to the Mediterranean coastal plains, the Sinai Peninsula, and the Saharo-Arabian deserts. Medicinal properties attributed to *T. hirsuta* include anti-inflammatory, cathartic, hydragogue, and expectorant effects, along with use as a remedy for pinworms, dental issues, eye diseases, and paralysis. Notably, it has shown inhibitory effects on hepatocellular carcinoma progression [19]. Aloe vera is a succulent plant in the Aloe genus. It is a perennial species thriving in arid, tropical, and semi-tropical climates. Its leaves contain bioactive compounds, such as acetylated mannans, polymannans, anthraquinone C-glycosides, anthrones, emodin, and lectins [20]. These phytochemicals contribute to A. vera’s antioxidant, anti-inflammatory, nutritional, and health-promoting effects. It is widely used in cosmetics, pharmaceuticals, and the food industry. Documented health benefits include wound healing, burn treatment, protection against X-rays, and activity against lung cancer, digestive disorders, diabetes, allergies, and AIDS. *Peganum harmala L*., also known as wild rue or harmel, is a perennial herbaceous plant in the Nitrariaceae family. It typically grows in saline soils in temperate desert and Mediterranean climates [21] and is considered a noxious weed and invasive species in some parts of the Western United States. Ethnopharmacological uses of *P. harmala* include treatments for fever, diarrhea, body pain, abortion, and subcutaneous tumors [21]. It exhibits notable anticholinesterase, anticancer, anti-inflammatory, antioxidant, antiparasitic, and antibacterial properties [22, 23]. Its bioactivity is largely due to β-carboline alkaloids, such as harmine, harmaline, harmalol, harman, and quinazoline derivatives, which also contribute to its toxicity in humans and animals [21]. Collectively, these plant species are aromatic, medicinal, or ornamental, though some are considered invasive and potentially harmful to ecosystems, agriculture, and human health. In this study, we utilized extracts from these plants as reducing agents in the green synthesis of ZnONPs. Particular attention was given to assessing their antimicrobial activity against bacterial and fungal strains, including *Staphylococcus aureus*, *Micrococcus luteus*, *Candida albicans*, and *Aspergillus flavus*. Furthermore, the bioavailability and pharmacokinetic properties of phenolic acids and flavonoids—identified via high-performance liquid chromatography coupled with mass spectrometry (HPLC-MS) in the extracts—were analyzed. Their molecular interactions with key biological receptors were also examined through computational modeling.

## Materials and methods

### Biosynthesis of ZnONPs

Plants of *Thymelaea hirsuta L*., *Aloe vera L*., *Retama monosperma L*., and *Peganum harmala L*. were collected in March 2020 from the regions of Beni Kdech and Bir Lahmar in the Medenine Governorate, Tunisia. The plant species were identified by Dr. Samir Tlahig (a specialist in botany) using the botanical guide La Flore de la Tunisie, Med-Checklist, and the online database Plants of the World Online (POWO). The aerial parts (leaves and shoots) of the plants were air-dried for five days at an ambient temperature of approximately 30–35 ^∘^C. Dried leaves (5 g) were ground using a mortar and pestle to obtain a fine powder, which was then homogenized in 50 mL of distilled water (1:10 w/v). The homogenates were filtered through ash-free filter paper and stored at 4 ^∘^C. For NP synthesis, a volume of each plant extract was mixed with 0.1 M zinc acetate dihydrate and incubated in a boiling water bath at 80 ^∘^C for 2 h. The resulting ZnONPs were collected by centrifugation at 8000 × *g* for 10 min. The supernatants were discarded, and the pellets were washed three times with distilled water to remove any remaining impurities. The synthesized ZnONPs were designated as follows: “Thymhirs.bio-ZnONP” for T. hirsuta-based ZnONPs, “Aloever.bio-ZnONP” for A. vera-based ZnONPs, “Retam.bio-ZnONP” for R. monosperma-based ZnONPs, and “Harm.bio-ZnONP” for P. harmala-based ZnONPs.

### Biophysical characterization

The structural properties of the biologically synthesized ZnONPs were characterized using a UV–visible spectrophotometer [14]. Particle size distribution and surface area were assessed by granulometry using a Malvern Mastersizer 2000 laser analyzer, within a measurement range of 0.02–2000 µm. Fourier Transform Infrared (FTIR) spectra were recorded using a Bruker Equinox 55 spectrometer over the wavelength range of 400–4000 cm^−1^ to identify the functional groups and phytochemicals present on the surface of the ZnONPs. Electron paramagnetic resonance (EPR) spectra were obtained using a Bruker ER-200D spectrometer operating at 9.8 GHz (X-band) with a modulation amplitude of 0.2 mT, a modulation frequency of 100 kHz, a sweep width of 210 mT, and microwave power of 63 mW. Data acquisition was performed at a power of 2 mW with a spectral range of 3200–3800 Gauss.

### Analysis of the flavonoids and phenolic compounds by liquid chromatography coupled to mass spectrometry (HPLC-MS)

Phenolic compounds were extracted by homogenizing dried leaf material in 80% methanol (1:10, w/v), followed by centrifugation at 10,000 × *g* for 20 min. The resulting supernatants were filtered through a 0.45 µm cellulose acetate filter (Millipore), and 20 µL of each extract was injected into a CTO-20 AC column for analysis by HPLC-MS, using a Shimadzu UFLC XR system (Kyoto, Japan). The system was equipped with an LC-20ADXR binary pump and a quadrupole 2020 mass detector. Separation was performed on an inertial ODS-4 C18 column (3 µm, 150 × 3.0 mm) maintained at 40 ^∘^C. The chromatographic run was carried out at a flow rate of 0.5 mL min^−1^ using a mobile phase consisting of solution A (5% methanol, 0.15% acetic acid) and solution B (50% acetonitrile, 0.15% acetic acid). The column was washed and equilibrated with 10% solution B for 45–50 min. The gradient elution program using solution B was as follows: 10%–20% from 0.01 to 14 min, 20%–55% from 14 to 27 min, 55%–100% from 27 to 37 min, and 100% from 37 to 45 min. The LC-ESI-MS conditions included a dissolving line temperature of 275 ^∘^C, a nebulizing gas flow of 1.50 mL min^−1^, a drying gas flow of 15.00 mL min^−1^, and a heat block temperature of 450 ^∘^C. Mass spectra were acquired in negative ion mode [M–H]^–^ using LabSolutions software. Identification and quantification of phenolic acids were performed by comparing the obtained mass spectra to those of reference standards for polyphenolic compounds (LGC and Sigma-Aldrich).

### Assay of the antibacterial activity

The bacterial strains used in this study included two Gram-positive strains—*Staphylococcus aureus* (ATCC 25923) and *Micrococcus luteus* (NCIMB 8166)—and two Gram-negative strains—*Salmonella enterica* serotype Typhimurium (ATCC 1408) and *Escherichia coli* (ATCC 35218). Bacterial cultures were grown on nutrient agar at 37 ^∘^C for 24 h. Selected colonies were suspended in 10 mL of sterile physiological saline and mixed for 5 min. Bacterial growth was monitored by measuring optical density (OD) at 600 nm and adjusted to an OD value of 0.5. A volume of 1 mL of each bacterial suspension was spread evenly onto Mueller–Hinton agar plates. Wells of 6 mm diameter were then filled with 100 µL of ZnONP solution (corresponding to a concentration of 10 × 10^3^ ppm or 0.1 mg of ZnONPs). The plates were incubated at 37 ^∘^C for 24 h, after which the diameter of the inhibition zones (in mm) was measured using a digital caliper. All antibacterial assays were performed in triplicate and under sterile conditions. Control assays were conducted using the individual plant extracts (*Thymelaea hirsuta L*., *Aloe vera L*., *Retama monosperma L*., and *Peganum harmala L*.), zinc acetate salt, and Gentamicin (10 µg/mL) as a standard antibiotic control.

### Activity against yeast growth

The yeast strains used in this study included *Candida albicans* (ATCC 90028), *Candida krusei* (ATCC 6258), and *Candida neoformans* (ATCC 14116). Cultures were initially grown on Sabouraud agar at 37 ^∘^C for 48 h. Selected colonies were then suspended in sterile physiological saline for preparation of yeast suspensions. Yeast growth was monitored by measuring the OD at 600 nm and adjusted to an OD value of 0.5. A 1 mL aliquot of each yeast suspension was spread onto Sabouraud agar plates and incubated at 37 ^∘^C for 30 min to allow initial adherence. Wells of 6 mm diameter were then filled with 100 µL of ZnONP solution (equivalent to 10 × 10^3^ ppm or 0.1 mg of ZnONPs). Plates were subsequently incubated at 37 ^∘^C for 48 h. After incubation, the diameters of the inhibition zones were measured using a digital caliper and recorded in millimeters. All antifungal assays were performed in triplicate and under sterile conditions. Control assays included treatments with plant extracts of *Thymelaea hirsuta L*., *Aloe vera L*., *Retama monosperma L*., and *Peganum harmala L*., as well as zinc acetate salt and Cycloheximide (10 µg/mL) as a positive control.

### Assay of the antifungal activity

The fungal strains *Aspergillus flavus* (15UA005), *Aspergillus niger* (15UA006), and *Aspergillus fumigatus* (ATCC 204305) were cultured on Sabouraud agar at 25 ^∘^C for seven days. Spores from each strain were individually harvested and suspended in peptone water, with the spore concentration adjusted to 10^6^ spores/mL. A volume of 1 mL from each spore suspension was inoculated onto Sabouraud agar plates and incubated at 25 ^∘^C for 30 min to allow initial fungal growth. Subsequently, 100 µL of ZnONP solution (corresponding to a concentration of 10 × 10^3^ ppm or 0.1 mg) was added into wells of 6 mm diameter on the agar surface. Plates were incubated at 25 ^∘^C for 48 h. After incubation, the diameters of the inhibition zones were measured using a digital caliper and recorded in millimeters. All antifungal assays were conducted in triplicate and under sterile conditions. Control treatments included the individual plant extracts of *Thymelaea hirsuta L*., *Aloe vera L*., *Retama monosperma L*., and *Peganum harmala L.*, as well as zinc acetate salt and Cycloheximide (10 µg/mL) as a positive control

### Computational modeling assay and interactions analyses

Phytochemicals, including phenolic acids and flavonoids identified in *Thymelaea hirsuta L.*, *Aloe vera L*., *Retama monosperma L*., and *Peganum harmala L*., were used in computational modeling to investigate their molecular interactions with key receptors involved in antimicrobial activity. The target receptors included tyrosyl-tRNA synthetase (TyrRS) from *Staphylococcus aureus*, secreted aspartic proteinase from *Candida albicans*, and wheat germ agglutinin. The 3D structures of the selected phytochemicals were either retrieved from the PubChem database or drawn using ChemDraw Pro 12.0. Receptor crystal structures were obtained from the RCSB Protein Data Bank: TyrRS from *S. aureus* (PDB ID: 1JIJ), secreted aspartic proteinase 1 from *C. albicans* (PDB ID: 2QZW), and wheat germ agglutinin (PDB ID: 2UVO). Prior to docking, receptors and ligands were prepared by removing water molecules, adding polar hydrogens and Kollman charges, and minimizing energy following established protocols [24–26]. Molecular docking was carried out using the AutoDock Vina software package. Grid boxes were determined for each receptor-ligand complex, and the CHARMm force field was applied to optimize molecular conformations and evaluate binding interactions [25, 27, 28].

### Bioavailability and pharmacokinetics

The bioavailability and pharmacokinetics of phenolic acids and flavonoids identified in *Thymelaea hirsuta L*., *Aloe vera L*., *Retama monosperma L*., and *Peganum harmala L*. were also evaluated, as previously described [27, 29]. Computational assessment of these parameters was based on ADMET profiling (Absorption, Distribution, Metabolism, Excretion, and Toxicity) [28, 30], along with the calculation of key physicochemical properties, including flexibility (Flex), unsaturation (Insatu), insolubility (Insolu), lipophilicity (Lipo), molecular size (Size), and polarity (Pola), using the SwissADMEweb tool.

### Statistical analysis

Statistical analyses were conducted using SPSS software (version 20). Data are presented as mean ± standard deviation (SD). Comparisons between types of ZnONPs were performed using one-way ANOVA at a significance level of α ═ 0.05, followed by Duncan’s multiple range post hoc test at 5% significance.

**Figure 1.**
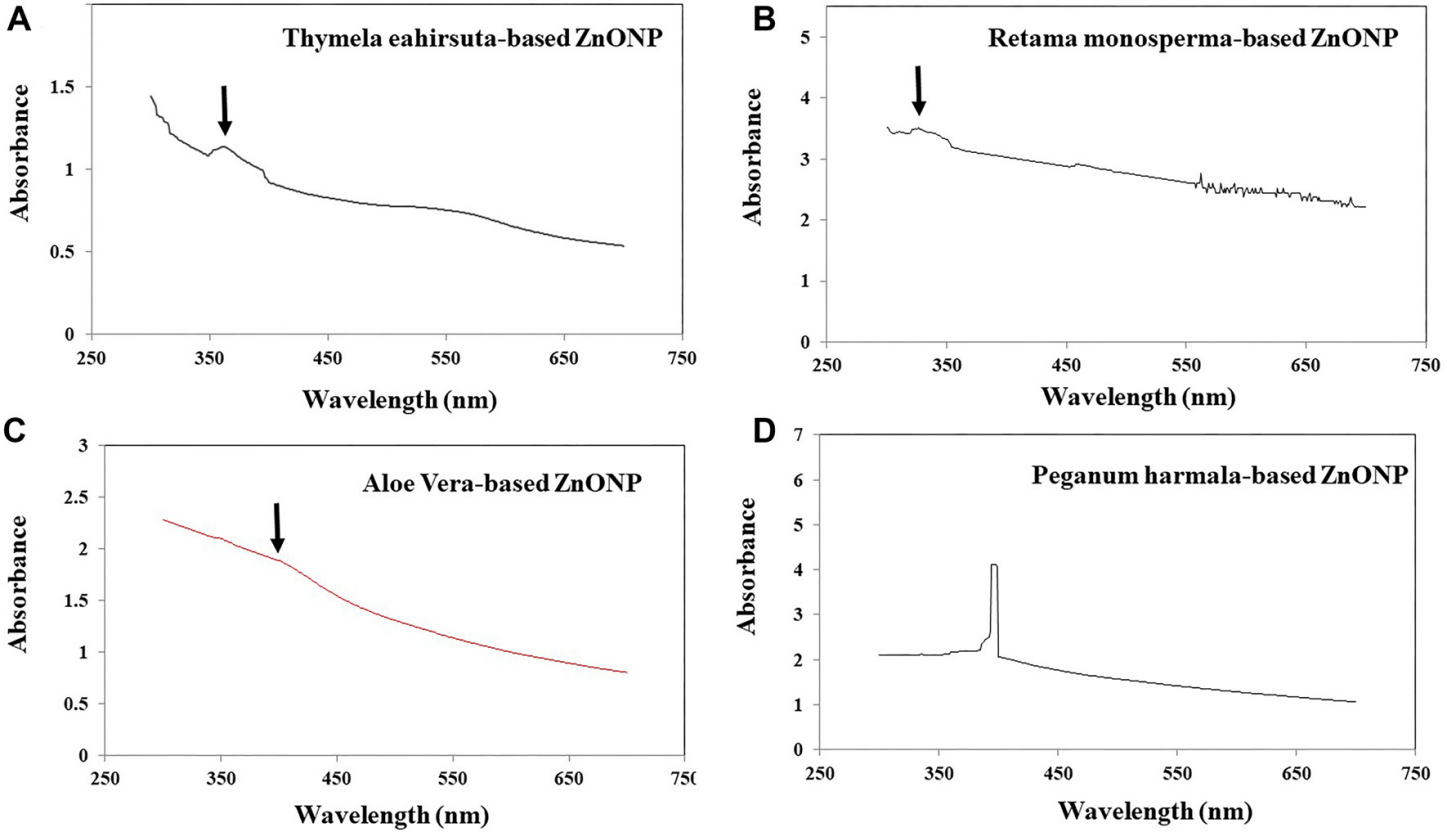
**UV–Vis spectra of plant-based ZnO nanoparticles.** UV–visible absorption spectra recorded in the range of 250–750 nm for zinc oxide nanoparticles synthesized using plant extracts. The spectra correspond to (A) *Thymelaea hirsuta* (Thymhirs.), (B) *Retama monosperma* (Retam.), (C) *Aloe vera* (Aloever.), and (D) *Peganum harmala* (Harm.). Each spectrum shows the characteristic absorption profile of the obtained nanoparticles, with arrows indicating the main absorption bands. ZnONP: Zinc oxide nanoparticle; Bio-ZnONP: Biosynthesized ZnONP; Thymhirs.: *Thymelaea hirsuta*; Aloever.: *Aloe vera*; Retam.: *Retama monosperma*; Harm.: *Peganum harmala*.

## Results

### Chemical and physical properties of nascent ZnONPs

**Figure 2.**
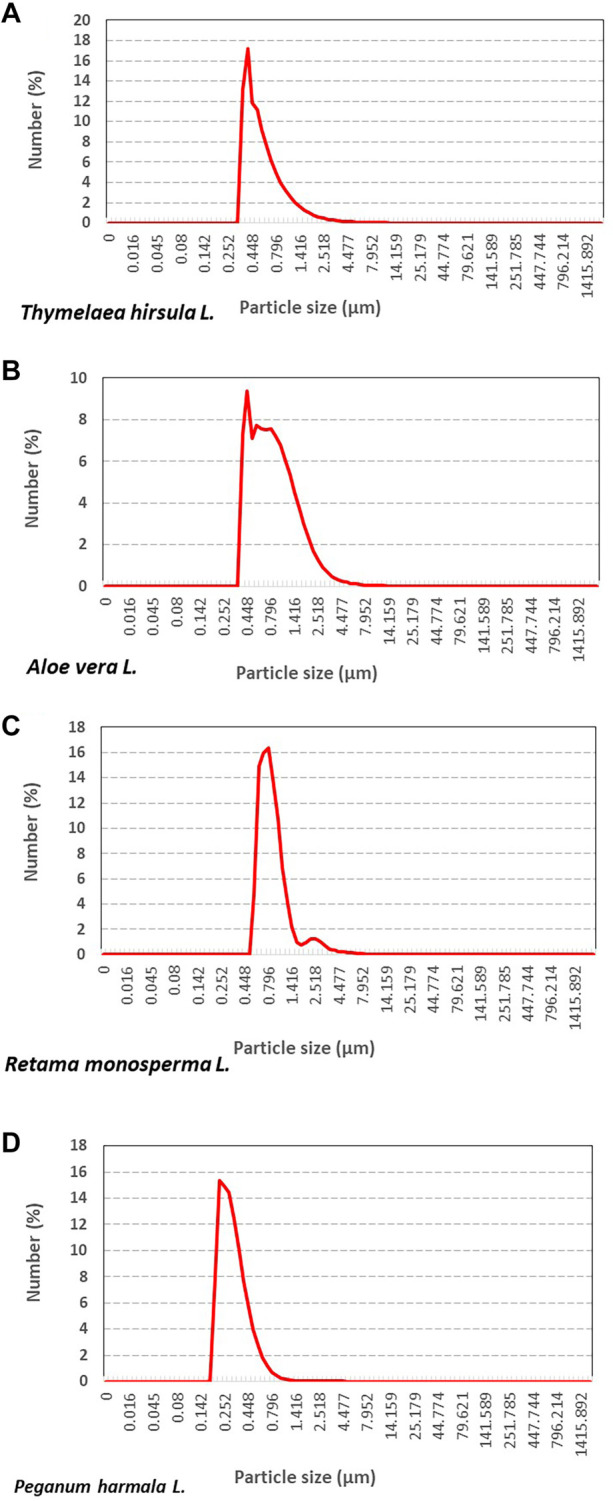
**Particle size distribution of plant-based ZnO nanoparticles.** Granulometric analysis of biosynthesized ZnO nanoparticles (bio-ZnONPs) obtained from (A) *Thymelaea hirsuta* (Thymhirs.), (B) *Aloe vera* (Aloever.), (C) *Retama monosperma* (Retam.), and (D) *Peganum harmala* (Harm.). Graphs show the percentage distribution of particle numbers as a function of particle size (µm). ZnONP: Zinc oxide nanoparticle; Bio-ZnONP: Biosynthesized ZnONP; Thymhirs.: *Thymelaea hirsuta*; Aloever.: *Aloe vera*; Retam.: *Retama monosperma*; Harm.: *Peganum harmala*.

*Thymelaea hirsuta L*., *Aloe vera L*., *Retama monosperma L*., and *Peganum harmala L*. were used to biosynthesize ZnONPs (bio-ZnONPs), designated as Thymhirs.bio-ZnONP, Aloever.bio-ZnONP, Retam.bio-ZnONP, and Harm.bio-ZnONP, respectively. The formation of bio-ZnONPs was confirmed by UV-visible spectroscopy, which showed characteristic absorption peaks in the range of 340–360 nm (Figure 1). Regarding particle size distribution (Figure 2), Thymhirs.bio-ZnONP exhibited average sizes of 392 nm at d(0.1) and 543 nm at d(0.5); Aloever.bio-ZnONP measured 410 nm at d(0.1) and 742 nm at d(0.5); Retam.bio-ZnONP showed 742 nm at d(0.1) and 980 nm at d(0.5); while Harm.bio-ZnONP exhibited smaller sizes, averaging 255 nm at d(0.1) and 348 nm at d(0.5) (Table 1). FTIR analysis revealed distinct peaks at 1634 cm^−1^ and in the range of 600–450 cm^−1^, corresponding to Zn–O stretching and deformation vibrations, respectively. As expected, metal oxide absorption bands typically appear in the 600–400 cm^−1^ region. Additionally, a broad peak around 3300 cm^−1^ was observed, indicating the presence of O–H stretching vibrations.

**Table 1:** Granulometry measurement of particle diameter of ZnONP

**Bio-ZnONP**	**d(0.1) µm**	**nm**	**d(0.5) µm**	**nm**	**d(0.9) µm**	**nm**
Thymhis.bio-ZnONP	0.392	392	0.543	543	1.212	1212
Aloever.bio-ZnONP	0.410	410	0.742	742	1.688	1688
Retam.bio-ZnONP	0.742	742	0.980	980	1.666	1666
Harm.bio-ZnONP	0.255	255	0.348	348	0.589	589

ZnONP: Zinc oxide nanoparticle; Bio-ZnONP: Biosynthesize ZnONP.

**Figure 3.**
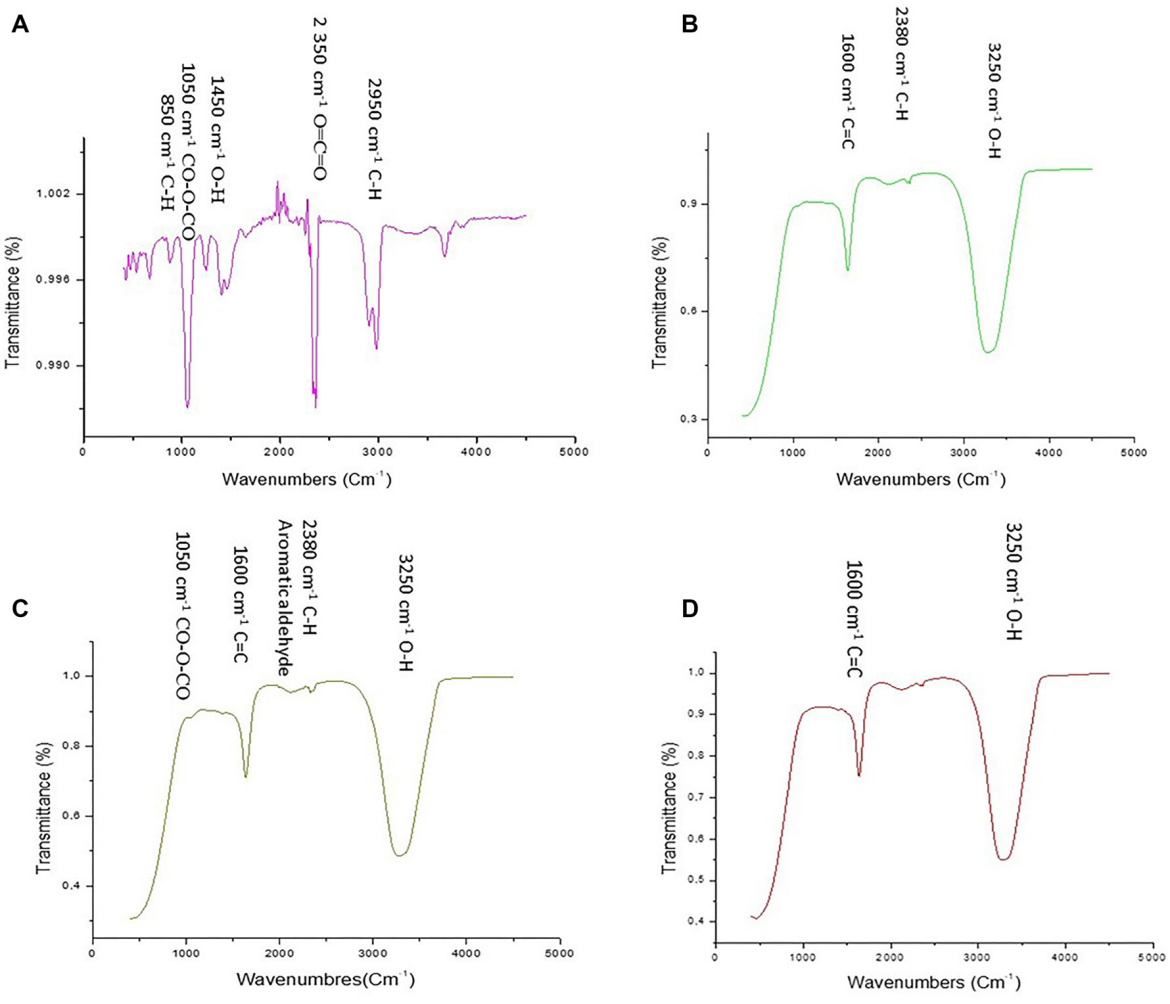
**FTIR spectra of plant-based ZnO nanoparticles.** FTIR spectra of bio-ZnONPs obtained using extracts of (A) *Thymelaea hirsuta* (Thymhirs.), (B) *Aloe vera* (Aloever.), (C) *Retama monosperma* (Retam.), and (D) *Peganum harmala* (Harm.). The spectra show characteristic bands associated with functional groups from plant extracts that act as reducing and stabilizing agents during nanoparticle formation. FTIR: Fourier-transform infrared; ZnONP: Zinc oxide nanoparticle; Bio-ZnONP: Biosynthesized ZnONP; Thymhirs.: *Thymelaea hirsuta*; Aloever.: *Aloe vera*; Retam.: *Retama monosperma*; Harm.: *Peganum harmala*.

The FTIR spectrum of *Thymhirs*.bio-ZnONP (Figure 3) showed a band in the range of 2950–2850 cm^−1^, corresponding to C–H stretching vibrations of alkane groups. A peak at 2350 cm^−1^ was attributed to the asymmetric stretching of O=C=O in carbon dioxide. An absorption peak at approximately 1450 cm^−1^ may indicate O–H bending of carboxylic acids. Additionally, peaks at 1050 cm^−1^ and 850 cm^−1^ corresponded to C–O–C stretching vibrations of anhydrides and C–H bending, respectively. The FTIR spectrum of *Aloever*.bio-ZnONP displayed absorption peaks between 3250–3400 cm^−1^, indicating O–H stretching of alcohol groups, and a peak at 1600 cm^−1^, corresponding to C=C stretching of α, β-unsaturated ketones. Similarly, *Retam*.bio-ZnONP exhibited a broad peak at 3250 cm^−1^, associated with O–H stretching of phenols or carboxylic acids, along with a peak at 1600 cm^−1^ due to C=C stretching of α, β-unsaturated ketones. These functional groups—including alkyl C–H, hydroxyl (–OH), and unsaturated C=C moieties—likely act as capping and stabilizing agents during ZnONP formation. Their presence may help prevent particle agglomeration and control NP growth.

### HPLC-MS results of phenolic acids and flavonoids

**Figure 4.**
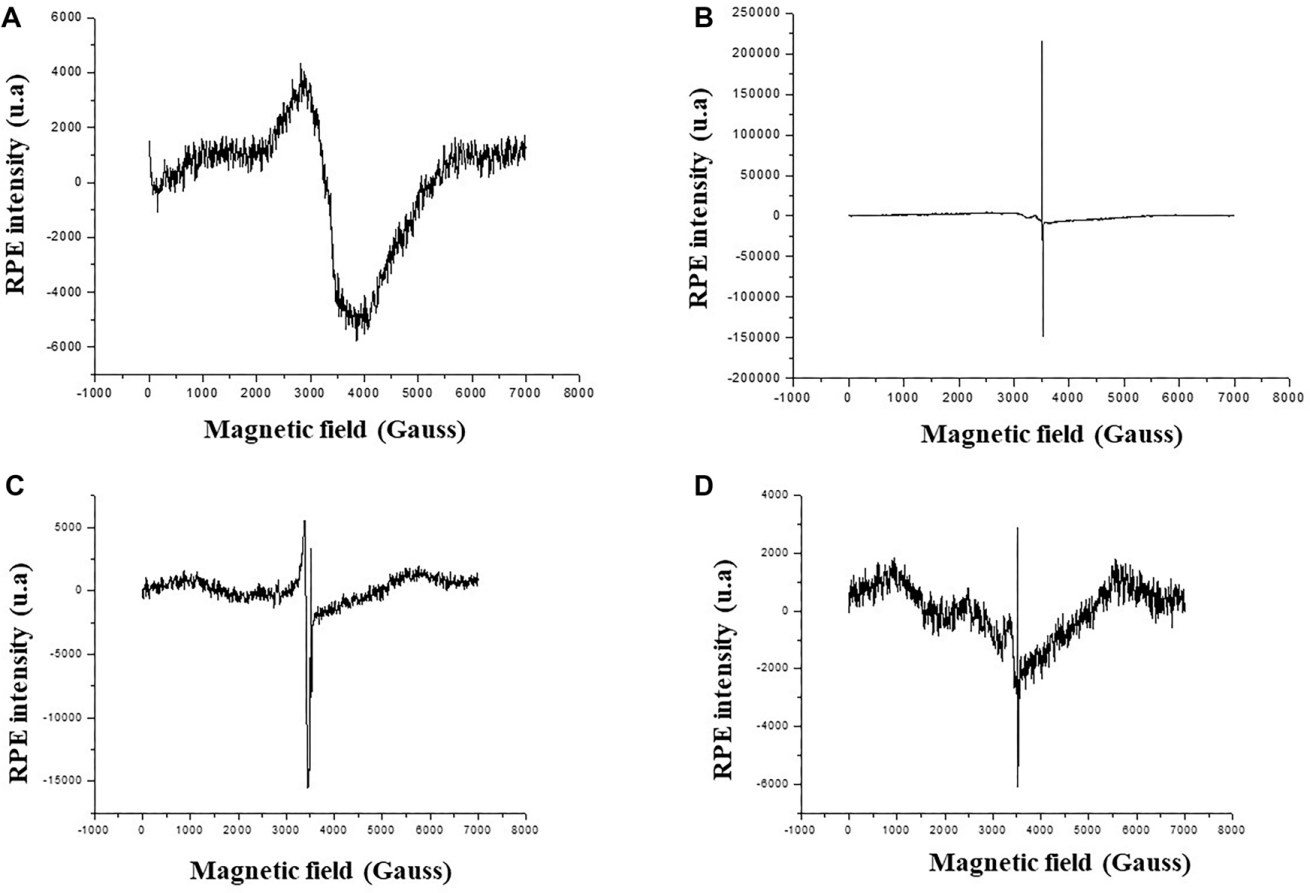
**EPR spectra of plant-based ZnO nanoparticles.** EPR spectra plotted as RPE intensity (a.u.) versus magnetic field (Gauss) for biosynthesized ZnO nanoparticles prepared from (A) *Thymelaea hirsuta* (Thymhirs.), (B) *Aloe vera* (Aloever.), (C) *Retama monosperma* (Retam.), and (D) *Peganum harmala* (Harm.). Each panel shows the recorded signal across the X-band field range with the principal resonance region centered near ∼3.4–3.6 kG. EPR: Electron paramagnetic resonance; ZnONP: Zinc oxide nanoparticle; Bio-ZnONP: Biosynthesized ZnONP; a.u.: Arbitrary units; Thymhirs.: *Thymelaea hirsuta*; Aloever.: *Aloe vera*; Retam.: *Retama monosperma*; Harm.: *Peganum harmala*.

The phenolic acids and flavonoids present in the plant extracts were evaluated using HPLC-MS (Table 2). The results revealed a diverse array of phenolic compounds that may function as reducing, stabilizing, and capping agents during the biosynthesis of ZnONPs. *Peganum harmala* exhibited the highest concentrations of quinic acid, gallic acid, trans-ferulic acid, rutin, luteolin-7-O-glucoside, 3,4-di-O-caffeoylquinic acid, caffeic acid, and naringenin. Some compounds were species-specific. For instance, *Thymelaea hirsuta* was particularly rich in quercetin, while *Aloe vera* contained significant amounts of protocatechuic acid, quercetin-3-O-rhamnoside, rosmarinic acid, catechin, and epicatechin. *A. vera* also exhibited the highest overall concentration of phenolic compounds, including rutin, caffeic acid, salviolinic acid, hyperoside (quercetin-3-O-galactoside), apigenin-7-O-glucoside, trans-cinnamic acid, chlorogenic acid, syringic acid, and cirsiliol. In contrast, *Retama monosperma L.* was primarily characterized by the presence of quinic acid, rutin, and 3,4-di-O-caffeoylquinic acid (Table 2). EPR analysis (Figure 4) revealed no detectable magnetic properties for *Thymhirs.bio*-ZnONP. In contrast, clear paramagnetic signals were observed for both *Aloever.bio*-ZnONP and Retam.bio-ZnONP. The EPR signal intensity, which reflects the total absorbed energy under resonance, indicated the presence of paramagnetic centers. A distinct signal was observed at *g* ═ 2.0124, which is attributed to VO–VZn clusters, confirming the paramagnetic nature of these ZnONPs.

**Table 2:** Qualitative and quantitative (ppm) of phenolic acids and flavonoids by HPLC-MS of extracts of *Thymelaea hirsuta* L., *Aloe vera* L., *Retama monosperma* L. and *Peganum harmala* L.

	**Phenolic compounds**	***Thymelaea hirsuta L.***	***Aloe vera L.***	***Retama monosperma L.***	***Peganum harmala L.***
1	Quinic acid	137.725	141.767	151.37	182.515
2	Gallic acid	0.153	0.106	0.051	0.162
3	Protocatechuic acid	0	2.84	0	0
4	p-coumaric acid	0.13	0	0.025	0.294
5	Trans-ferulic acid	0.014	0.08	0	0.23
6	Rutin	0.225	0.53	0.194	0.452
7	Luteolin-7-O-glucoside	0	2.205	0	4.414
8	3,4-di-O-caffeoyquinic acid	0	0	0.151	0.591
9	Caffeic acid	0	1.007	0	0.114
10	Salviolinic acid	0	1.167	0	0
11	Quercetin	0.022	0	0	0
12	Quercetin (quercetin-3-o-rhamonosic)	0	6.377	0	0
13	Hyperoside (quercetin-3-o-galactoside)	0	0.556	0.157	0
14	Naringin	0	2.623	0	0
15	Naringenin	0	1.321	0	6.978
16	Apegenin-7-o-glucoside	0.262	4.282	0.017	2.616
17	Apegenin	0	0.47	0	5.409
18	Rosmarinic acid	0	0.099	0	0
19	Acacetin	0	0	0	0
20	Trans cinnamic acid	0	0.309	0	0.06
21	Cirsiliol	0.712	11.452	0.287	0.734
22	(+)-Catechin	0	9.723	0	0
23	Epicatechin	0	0.878	0	0
24	Chlorogenic acid	0	171.64	2.866	0
25	Syringic acid	0	0.366	0	0.076

### Biological activities

Table 3 presents the antimicrobial activity of the synthesized bio-ZnONPs against a range of microorganisms, including bacterial, yeast, and fungal strains. The disk diffusion method, used for this evaluation, is a qualitative or semi-quantitative technique. Its sensitivity depends on several factors, including the diffusion rate of the compound through the agar, its concentration, and the susceptibility of the target microbe. A zero inhibition zone in this assay does not necessarily indicate a complete lack of antimicrobial activity; rather, it suggests that any effect is below the detection threshold of the method used. In this study, inhibition zones were interpreted as follows: diameters ≥15 mm were considered indicative of strong antimicrobial activity; diameters between 10 and 14 mm were classified as moderate activity; and diameters <10 mm were considered weak activity. Among the bacterial strains tested, the strongest antibacterial activity was observed against *Staphylococcus aureus* ATCC 25923, with *Thymhirs*.bio-ZnONP producing an inhibition zone of 15 ± 1.41 mm. The highest overall antibacterial effect was recorded for *Aloever*.bio-ZnONP against *Micrococcus luteus* NCIMB 8166, with a zone of 24 ± 0.00 mm.

**Table 3:** Antimicrobial activities (Inhibition diameter, mm) of “*Thymhirs*.bio-ZnONP,” “*Aloever*.bio-ZnONP,” “*Retam*. bio-ZnONP” and “*harm*. Bio-ZnONP”

**Bacterial strains**	***Thymhirs*.bio-ZnONP**	***Aloever.*bio-ZnONP**	***Retam*.bio-ZnONP**	***harm.*bio-ZnONP**	**Plant extracts**			
	**Antibacterial activity (Inhibition diameter, mm)**				***T. hir.***	***A. ver.***	***R. mon.***	***P. har.***
*Staphylococcus aureus*	15 ± 1.41 ^a^	13 ± 0.71 ^ab^	11 ± 0.71 ^b^	13 ± 0.71 ^ab^	0 ± 0 ^a^	0 ± 0 ^b^	0.33 ± 0.57 ^a^	23 ± 1.00 ^a^
*Micrococcus luteus*	13 ± 0.71 ^b^	24 ± 0.00 ^a^	13 ± 0.71 ^b^	12 ± 0.71 ^c^	0 ± 0 ^a^	0 ± 0 ^b^	1 ± 1.00 ^a^	10 ± 1.00 ^a^
*Salmonella ent. ser. Typh.*	0 ^b^	0 ^b^	11 ± 0.00 ^a^	0 ^b^	0 ± 0 ^a^	0 ± 0 ^b^	1.33 ± 0.58 ^a^	11.33 ± 1.53 ^a^
*Escherichia coli*	0	0	0	0	0 ± 0 ^a^	15 ± 1.00 ^a^	11 ± 1.00 ^a^	11 ± 1.00 ^a^
*Yeast strains*	*Activity against yeast growth (Inhibition diameter, mm)*							
*Candida albicans*	14 ± 0.71 ^a^	14 ± 0.71 ^a^	13 ± 0.00 ^b^	0 ^c^	11 ± 1.00 ^a^	7.67 ± 2.51 ^a^	8.00 ± 1.00 ^a^	0.67 ± 1.00 ^a^
*Candida krusei*	0 ^b^	12 ± 0.71 ^a^	0 ^b^	0 ^b^	0.0 ± 0.0 ^b^	8.0 ± 1.73 ^a^	0.00 ± 0.00 ^b^	0.00 ± 0.00 ^a^
*Candida neoformans*	0 ^c^	11 ± 0.00 ^b^	0 ^c^	16 ± 0.71 ^a^	0.0 ± 0.0 ^b^	8.0 ± 1.00 ^a^	0.00 ± 0.00 ^b^	11.33 ± 0.58 ^a^
*Fungal strains*	*Antifungal activity (Inhibition diameter, mm)*							
*Aspergillus flavus*	16 ± 0.71 ^b^	16 ± 1.41 ^b^	17 ± 0.71 ^a^	14 ± 0.71 ^c^	10.00 ± 1.00 ^a^	11.67 ± 1.15 ^ab^	8.00 ± 1.00 ^a^	11.00 ± 1.00 ^a^
*Aspergillus niger*	0 ^c^	16 ± 1.41 ^a^	0 ^c^	15 ± 0.00 ^b^	0.67 ± 0.57 ^b^	12.00 ± 2.00 ^a^	8.00 ± 1.00 ^a^	10.00 ± 1.00 ^b^
*Aspergillus fumigatus*	12 ± 0.00 ^b^	12 ± 0.71 ^b^	0 ^c^	16 ± 0.71 ^a^	9.00 ± 1.00 ^a^	7.00 ± 1.73 ^b^	8.00 ± 1.00 ^a^	11.00 ± 1.73 ^a^

Bacterial strains consisted of *Staphylococcus aureus* (ATCC 25 923), *Micrococcus luteus* (NCIMB 8166), *Salmonella enterica serotype Typhimurium* (ATCC 1408) and *Escherichia coli* (ATCC35218). Yeast strains consisted of *Candida albicans* (ATCC90028), *Candida krusei* (ATCC6258) and *Candida neoformans* (ATCC14116). Fungal strains consisted of *Aspergillus flavus* (15UA005), *Aspergillus niger* (15UA006) and *Aspergillus fumigatus* (ATCC204305). *T. hir., A. ver., R. mon.,* and *P. har.* referred to the assays using plant extracts, respectively, of *Thymelaea hirsuta L., Aloe vera L., Retama monosperma L., and Peganum harmala L*. The significance of the difference between microbial strains was determined using ANOVA (α ═ 0.05). Letters denote statistical difference between the types of ZnONPs, using the Duncan test (α ═ 0.05). ZnONP: Zinc oxide nanoparticle; Bio-ZnONP: Biosynthesize ZnONP.

*Retam*.bio-ZnONP exhibited inhibitory activity against *Staphylococcus aureus* (11 ± 0.71 mm), *Micrococcus luteus* (13 ± 0.71 mm), and *Salmonella enterica* serotype *Typhimurium* (11 ± 0.00 mm). Similarly, Harm.bio-ZnONP inhibited the growth of *S. aureus* (13 ± 0.71 mm) and *M. luteus* (12 ± 0.71 mm), but showed no activity against *S. enterica* or *Escherichia coli* ATCC 35218. Regarding yeast strains, *Thymhirs*.bio-ZnONP showed inhibitory activity only against *Candida albicans* ATCC 90028. In contrast, *Aloever.bio*-ZnONP exhibited broader antifungal activity, effectively inhibiting all three tested strains—*C. albicans* (14 ± 0.71 mm), *Candida krusei* (12 ± 0.71 mm), and *Candida neoformans* (11 ± 0.00 mm). *Retam.*bio-ZnONP also inhibited *C. albicans* (13 ± 0.00 mm), while Harm.bio-ZnONP was active only against *C. neoformans*, with an inhibition zone of 16 ± 0.71 mm. Interestingly, Harm.bio-ZnONP demonstrated a much stronger antifungal effect against filamentous fungi. It showed significant inhibitory activity against all Aspergillus species tested, with increasing effectiveness in the following order: *A. flavus* <; *A. niger* <; *A. fumigatus*. *Thymhirs*.bio-ZnONP also inhibited *Aspergillus flavus* and *Aspergillus fumigatus*, with inhibition zones of 16 ± 0.71 mm and 12 ± 0.00 mm, respectively. *Aloever*.bio-ZnONP displayed antifungal activity against all Aspergillus strains, with the strongest effects observed against *A. flavus* and *A. fumigatus*. Additionally, *Retam*.bio-ZnONP effectively inhibited *A. flavus*, producing an inhibition zone of 17 ± 0.71 mm (Table 3).

We also compared the antimicrobial effects of the plant extracts with those of their corresponding ZnONPs (Table 3). The results showed either no detectable inhibition (0 mm) or weak antibacterial activity (inhibition zone <10 mm) for most of the plant extracts. An exception was observed for *Peganum harmala*, which demonstrated strong antibacterial activity against *Staphylococcus aureus* (15 ± 1.00 mm) and moderate inhibition across all tested bacterial, yeast, and fungal strains (Table 3). Similarly, zinc acetate salt exhibited weak antimicrobial activity (inhibition zones <10 mm) against most of the tested microbial strains (Table 4), with the exception of moderate inhibitory effects observed against *S. aureus* and *Micrococcus luteus* (Table 4).

**Table 4:** Control assays of the antimicrobial activities (Inhibition diameter, mm) of Zinc acetate salt, and commercial antibiotics; Gentamicin (10 µg/mL) and Cycloheximide (10 µg/mL)

	**Inhibitory diameter (mm)**		
	**Zinc acetate salt**	**Gentamicin**	**Cycloheximide**
*Bacterial strains*			
*Staphylococcus aureus*	11.33 ± 1.52 ^a^	25 ± 2.00 ^a^	*
*Micrococcus luteus*	11 ± 0.577 ^a^	22 ± 2.00 ^b^	*
*Salmonella en. ser. Typh.*	4 ± 0.00 ^c^	15.66 ± 1.52 ^ab^	*
*Escherichia coli*	10.67 ± 2.08 ^b^	22 ± 2.00 ^b^	*
*Yeast strains*			
*Candida albicans*	1.66 ± 0.57 ^ab^	*	21 ± 1.0 ^a^
*Candida krusei*	2.33 ± 0.57 ^a^	*	19.66 ± 1.52 ^b^
*Candida neoformans*	0.66 ± 0.57 ^c^	*	16.66 ± 2.89 ^c^
*Fungal strains*			
*Aspergillus flavus*	7 ± 1.0 ^a^	*	21.33 ± 2.08 ^ab^
*Aspergillus niger*	2 ± 1.0 ^b^	*	22.66 ± 1.15 ^a^
*Aspergillus fumigatus*	3 ± 1.0 ^b^	*	19 ± 2.00 ^b^

Bacterial strains consisted of *Staphylococcus aureus* (ATCC 25 923), *Micrococcus luteus* (NCIMB 8166), *Salmonella enterica serotype Typhimurium* (ATCC 1408) and *Escherichia coli* (ATCC35218). Yeast strains consisted of *Candida albicans* (ATCC90028), *Candida krusei* (ATCC6258) and *Candida neoformans* (ATCC14116). Fungal strains consisted of *Aspergillus flavus* (15UA005), *Aspergillus niger* (15UA006) and *Aspergillus fumigatus* (ATCC204305). The significance of the difference between microbial strains was determined using ANOVA (α ═ 0.05). Letters denote statistical difference between the types of ZnONPs, using the Duncan test (α ═ 0.05). ZnONP: Zinc oxide nanoparticle.

### Computational findings: pharmacokinetics and molecular interactions

**Figure 5.**
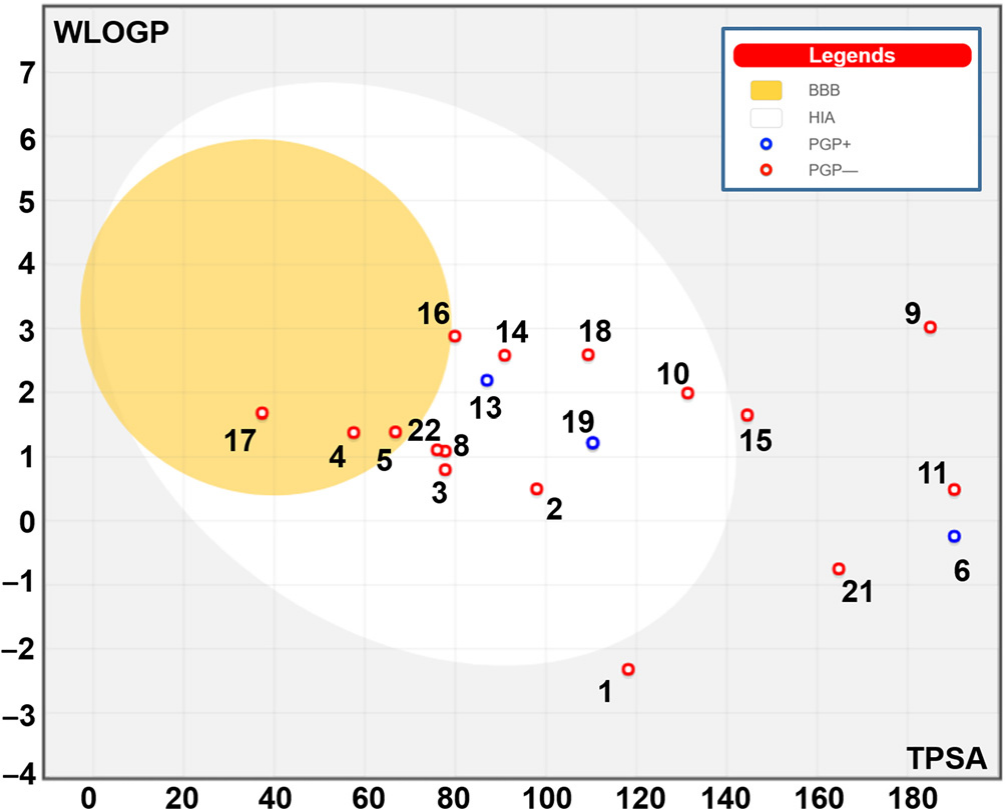
**BOILED-Egg model of the identified phytochemicals.** Plot of the BOILED-Egg model showing the position of compounds 1–25 on a WLOGP (lipophilicity) vs. TPSA (topological polar surface area) map. The yellow (yolk) region denotes the physicochemical space associated with BBB permeation, while the white (egg white) region indicates predicted high gastrointestinal absorption. Red and blue markers indicate predicted P-gp non-substrates and substrates, respectively. BBB: Blood–brain barrier; GI: Gastrointestinal; P-gp: P-glycoprotein; TPSA: Topological polar surface area; WLOGP: Wildman–Crippen logP.

The lipophilicity, drug-likeness, and pharmacokinetic properties of the phenolic acids and flavonoids identified in *Thymelaea hirsuta L*., *Aloe vera L*., *Retama monosperma L*., and *Peganum harmala L*. are presented in Table 5. Most of these phytochemicals comply with Lipinski’s Rule of Five, indicating favorable drug-likeness profiles. Based on key parameters—including flexibility (Flex), unsaturation (Insatu), insolubility (Insolu), lipophilicity (Lipo), molecular size (Size), and polarity (Pola)—the compounds demonstrated acceptable oral bioavailability scores, ranging from 0.11 to 0.85, supporting their potential bioactive properties. Pharmacokinetic predictions included blood–brain barrier (BBB) permeability and gastrointestinal (GI) absorption, both of which were incorporated into the design of the BOILED-Egg model (Figure 5). Most of the identified compounds were not P-glycoprotein (P-gp) substrates and exhibited acceptable skin permeability, with Log Kp values ranging from −5.66 to −9.15. Additionally, Table 4 shows that while only a few of the studied compounds inhibited individual cytochrome P450 enzymes (CYPs), none were predicted to inhibit CYP2C19. The synthetic accessibility scores ranged from 1.07 to 5.28, indicating varying degrees of ease for chemical synthesis.

**Table 5:** Lipophilicity, druglikeness, and pharmacokinetics of the phenolic acids and flavonoids (1–25) as identified by HPLC-MS extracts of *Thymelaea hirsuta* L., *Aloe vera* L., *Retama monosperma* L. and *Peganum harmala*. L. based on their ADMET (for absorption, distribution, metabolism, excretion and toxicity) properties

**Entry**	**1**	**2**	**3**	**4**	**5**	**7**	**8**	**9**	**10**	**11**	**12**	**13**	**15**	**17**	**18**	**19**	**20**	**21**	**22**	**23**	**24**	**25**
	*Lipophilicity & physicochemical properties*																					
**TPSA**	118.22	97.99	77.76	57.53	66.76	190.28	211.28	77.76	184.98	131.36	190.28	210.51	86.99	90.9	144.52	79.9	37.3	109.36	110.38	110.38	164.75	75.99
**Log Po/w (iLOGP)**	0.37	0.21	0.66	0.95	1.62	1.76	1.32	0.97	2.19	1.63	1.6	1.45	1.75	1.89	1.48	2.56	1.55	2.46	1.33	1.47	0.87	1.54
**Consensus Log Po/w**	−1.66	0.21	0.65	1.26	1.36	0.15	0.8	0.93	2.66	1.23	0.22	−0.38	1.84	2.11	1.58	2.52	1.79	2.13	0.83	0.85	−0.39	0.99
**Log S (ESOL) solubility**	0.53	−1.64	−1.86	−2.02	−2.11	−3.65	−3.65	−1.89	−5.15	−3.16	−3.33	−3.04	−3.49	−3.94	−3.44	−4.14	−2.37	−4.12	−2.22	−2.22	−1.62	−1.84
	*Druglikeness & medicinal chemistry*																					
**Lipinski**	Yes	Yes	Yes	Yes	Yes	No	No	Yes	Yes	Yes	No	No	Yes	Yes	Yes	Yes	Yes	Yes	Yes	Yes	Yes	Yes
**Biovailability score**	0.56	0.56	0.56	0.85	0.85	0.17	0.11	0.56	0.11	0.55	0.17	0.17	0.55	0.55	0.56	0.55	0.85	0.55	0.55	0.55	0.11	0.56
**Leadlikeness**	1	1	1	1	1	1	2	1	3	0	1	1	0	0	1	0	1	0	0	0	1	1
**Synthetic accessibility**	3.34	1.22	1.07	1.61	1.93	5.17	4.86	1.81	4.18	3.23	5.28	5.32	3.01	2.96	3.38	2.98	1.67	3.32	3.5	3.5	4.16	1.7
	*Pharmacokinetics*																					
**GI absorption**	Low	High	High	High	High	Low	Low	High	Low	High	Low	Low	High	High	Low	High	High	High	High	High	Low	High
**BBB permeant**	No	No	No	Yes	Yes	No	No	No	No	No	No	No	No	No	No	No	Yes	No	No	No	No	No
**P-gp substrate**	No	No	No	No	No	Yes	Yes	No	No	No	No	No	Yes	No	No	No	No	No	Yes	Yes	No	No
**CYP1A2**	No	No	No	No	No	No	No	No	No	Yes	No	No	Yes	Yes	No	Yes	No	Yes	No	No	No	No
**CYP2C19**	No	No	No	No	No	No	No	No	No	No	No	No	No	No	No	No	No	No	No	No	No	No
**CYP2C9**	No	No	No	No	No	No	No	No	Yes	No	No	No	No	No	No	Yes	No	Yes	No	No	No	No
**CYP2D6**	No	No	No	No	No	No	No	No	No	Yes	No	No	No	Yes	No	Yes	No	Yes	No	No	No	No
**CYP3A4**	No	Yes	Yes	No	No	No	No	No	No	Yes	No	No	Yes	Yes	No	Yes	No	Yes	No	No	No	No
**Log Kp (skin permeation)**	−9.15	−6.84	−6.42	−6.26	−6.41	−8	−8.37	−6.58	−6.53	−7.05	−8.42	−8.88	−6.17	−5.8	−6.82	−5.66	−5.69	−6.14	−7.82	−7.82	−8.76	−6.77

TPSA: Topological polar surface area; GI: Gastro-intestinal; BBB: Blood-brain-barrier; P-gp: P-glycoprotein; CYP: Cytochrome P450; HPLC-MS: High-performance liquid chromatography coupled with mass spectrometry.

**Figure 6.**
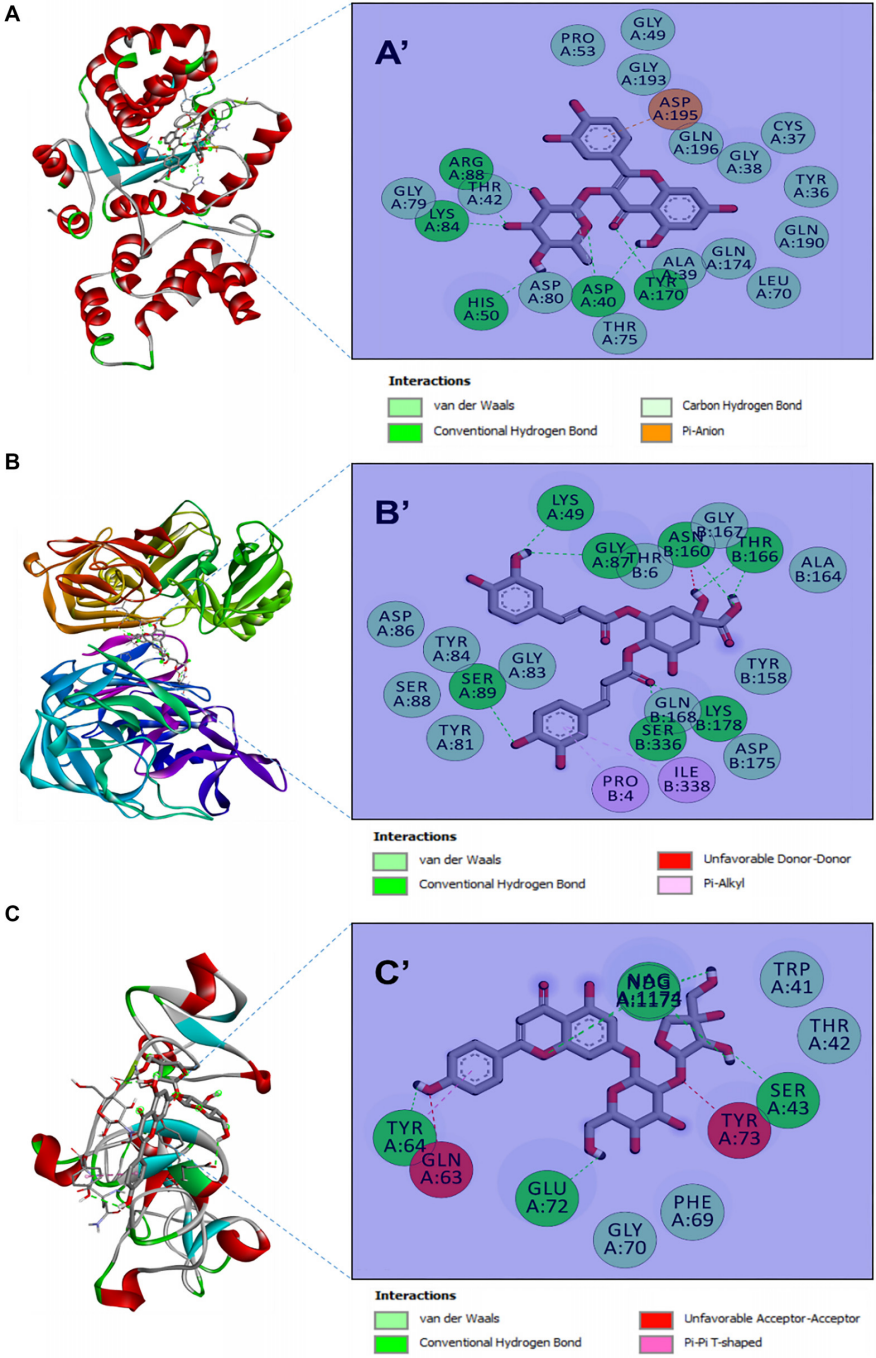
**Top-ranked molecular docking complexes and interaction maps.** 3D ribbon views of target proteins with docked phytochemicals (A–C) and their corresponding 2D interaction diagrams (A’–C’). Shown are the best-scoring ligands: compound 12 bound to 1JIJ (TyrRS) (A, A’), compound 8 bound to 2QZW (secreted aspartic proteinase) (B, B’), and compound 16 bound to 2UVO (wheat germ agglutinin) (C, C’). The 2D maps highlight key contacts (conventional H-bonds, Π–Π/Π–alkyl, carbon H-bonds, and van der Waals) that stabilize each receptor–ligand complex; these three ligands showed the strongest binding affinities among the tested compounds. TyrRS: Tyrosyl-tRNA synthetase; H-bond: Hydrogen bond; Π–Π/Π–alkyl: Aromatic stacking/alkyl–Π interaction.

All the studied phytochemicals were predicted to exhibit negative binding affinities toward the three target receptors—1JIJ, 2QZW, and 2UVO—indicating favorable molecular interactions (Table 6). The strongest binding affinities were −10.5 kcal/mol for 1JIJ, −10.0 kcal/mol for 2QZW, and −8.4 kcal/mol for 2UVO, corresponding to phytochemicals Nos. 12, 8, and 16, respectively (Tables 6 and 7). Several phytochemicals formed strong molecular interactions with the target receptors, including up to nine conventional hydrogen bonds. For example, compound No. 8 established nine hydrogen bonds, supported by additional van der Waals and π-alkyl interactions (Figure 6). These interactions involved residues LYS49, SER89, ASN160, LYS178, SER336, GLY87, PRO4, ILE338, and THR166. Notably, THR166 formed three hydrogen bonds with compound No. 8 and was the closest interacting residue, at a distance of 2.150 Å.

**Table 6:** Binding energy of the phenolic acids and flavonoids (1–25) as identified by HPLC-MS extracts of *Thymelaea hirsuta* L., *Aloe vera* L., *Retama monosperma* L. and *Peganum harmala*. L. and the 3 targeted receptors: 1JIJ, 2QZW and 2UVO for TyrRS from *Staphylococcus aureus*, aspartic proteinase from *Candia albicans*, and wheat germ agglutinin (2UVO), respectively

**Receptor/Ligand**	**Binding energy (kcal/mol)**		
	**1JIJ**	**2QZW**	**2UVO**
1	−7.1	−6.1	−6.0
2	−7.3	−5.8	−5.5
3	−7.0	−5.7	−5.8
4	−6.5	−6.0	−5.2
5	−6.9	−6.4	−5.1
6	−9.5	−8.8	−7.6
7	−9.5	−9.9	−7.8
8	−9.4	−10.0	−6.9
9	−7.3	−6.4	−6.0
10	−9.7	−9.9	−7.4
11	−9.9	−8.2	−7.2
12	−10.5	−8.4	−7.4
13	−9.4	−8.8	−7.4
14	−8.6	−8.8	−8.4
15	−9.4	−8.2	−7.1
16	−9.1	−9.1	−8.4
17	−9.5	−8.2	−7.1
18	−7.6	−8.4	−6.5
19	−9.5	−8.3	−7.0
20	−6.3	−6.0	−5.4
21	−8.7	−8.5	−7.1
22	−9.2	−8.1	−7.1
23	−9.1	−8.4	−7.4
24	−9.0	−8.8	−6.8
25	−7.0	−5.9	−5.2

HPLC-MS: High-performance liquid chromatography coupled with mass spectrometry; TyRS: Tyrosyl-tRNA Synthetase.

**Table 7:** Interactions, bond category and closest interacting residues for the best identified compounds of the phenolic acids and flavonoids (1–25) as identified by HPLC-MS extracts of *Thymelaea hirsuta* L., *Aloe vera* L., *Retama monosperma* L. and *Peganum harmala*. L. with the targeted receptors: 1JIJ, 2QZW and 2UVO for TyrRS from *Staphylococcus aureus*, aspartic proteinase from *Candia albicans*, and wheat germ agglutinin, respectively

**Compound No.**	**No. H-Bond**	**Closest interacting residues**	
		**Residue** **(Letters & ID)**	**Distance to closest** **interacting** **residue (Å)**
*TyrRS from Staphylococcus aureus (pdb id: 1JIJ)*			
12 (−10.5)	8	**ASP40, LYS84, ARG88, ARG88, TYR170, HIS50, ASP40**, ASP40, ASP195	LYS84:HZ1 (1.875)
11 (−9.9)	5	**LYS84, LYS84, ASN124, ASP195, ASP177**, LEU70	ASP177:OD1 (1.774)
10 (−9.7)	9	**LYS84, LYS84, LYS84, LYS84, GLY193, GLN196, ASP195, THR75**, GLY192, ASP40, ASP195, ALA39	LYS84:HZ2 (2.195)
*Aspartic proteinase from Candia albicans (pdb id: 2QZW)*			
8 (−10.0)	9	**LYS49, SER89, ASN160, LYS178, SER336, GLY87, THR166, THR166, THR166**, PRO4, ILE338	THR166:O (2.150)
7 (−9.9)	9	**TYR81, TYR81, SER89, GLN168, SER336, SER334, SER88, THR6, GLY83**, ILE338, GLY102;GLY103, PRO4, PRO4, PRO4	SER88:O (2.142)
10 (−9.9)	8	**TYR225, SER301, SER301, SER334, GLY34, GLU193, ASP86, GLY220**, ASP86, THR221, TYR225, ALA335	SER301:HG (2.173)
*Wheat germ agglutinin (pdb id: 2UVO)*			
16 (−8.4)	9	**SER43, GLU72**, **TYR64, NDG1173, NAG1174, NDG1173, NAG1174, NDG1173, NAG1174,** TYR64	NAG1174:O4 (1.890)
14 (−8.4)	5	**TRP41, GLN59, CYS40, SER43, GLN59**, **NDG1173, NAG1174, NDG1173, NAG1174,** GLU72	TRP41:O (1.817)
7 (−7.8)	5	**SER43, TRP41, GLU72**, **NDG1173, NAG1174, NDG1173, NAG1174,** NDG1173, NAG1174, TYR64	NAG1174:O4 (2.303)

Bold residues: Interacting with Conventional H-Bonds. HPLC-MS: High-performance liquid chromatography coupled with mass spectrometry; TyRS: Tyrosyl-tRNA Synthetase.

## Discussion

The biosynthesis approach employed in this study involved the use of zinc acetate as a precursor and plant extracts as sources of reducing, stabilizing, and capping agents. The novelty of this work lies in the utilization of desert plant species with medicinal or invasive characteristics, specifically *Thymelaea hirsuta L*., *Aloe vera L*., *Retama monosperma L*., and *Peganum harmala L*. The resulting ZnONPs (bio-ZnONPs) were designated as *Thymhirs*.bio-ZnONP, *Aloever*.bio-ZnONP, *Retam*.bio-ZnONP, and *Harm*.bio-ZnONP, respectively. The formation of bio-ZnONPs was confirmed by UV-visible spectroscopy, with all samples showing characteristic absorption peaks in the range of 340–400 nm, indicative of nanosized ZnO particles. FTIR analysis further confirmed the presence of functional groups, such as alcohols, phenols, amines, and carboxylic acids, which are involved in the reduction and stabilization of ZnONPs. These phytochemicals interact with zinc ions, promoting NP formation and providing surface stabilization to prevent agglomeration. HPLC-MS analysis revealed a diverse profile of phenolic acids and flavonoids in the plant extracts, including quinic acid, gallic acid, trans-ferulic acid, rutin, luteolin-7-O-glucoside, 3,4-di-O-caffeoylquinic acid, caffeic acid, naringenin, quercetin, protocatechuic acid, quercetin-3-O-rhamnoside, rosmarinic acid, catechin, epicatechin, salviolinic acid, hyperoside (quercetin-3-O-galactoside), apigenin-7-O-glucoside, trans-cinnamic acid, chlorogenic acid, syringic acid, and cirsiliol. The variation in phytochemical composition across these plant species plays a key role in influencing the structural stability, reactivity, and potential applications of the resulting ZnONPs. Additionally, other bioactive compounds, such as tannins, alkaloids, terpenoids, sugars, ketones, and steroids are likely involved in NP stabilization by acting as natural capping, reducing, and stabilizing agents. These compounds form an organic coating on the NP surface, enhancing colloidal stability, reducing particle aggregation, and improving dispersibility in various solvents [31, 32]. Previous studies involving other plant species—such as *Cannabis sativa*, *Ceratonia siliqua*, and *Artemisia vulgaris*—have also highlighted the role of flavonoids, steroids, coumarins, alkaloids, glycosides, anthraquinones, monoterpenes, diterpenes, and phenolic compounds in the biosynthesis and stabilization of ZnONPs [12–14]. Phenolic acids and flavonoids are particularly important due to their multifunctional roles in NP synthesis. Their phenolic hydroxyl groups can donate electrons, reducing Zn^2+^ ions to ZnO, while their aromatic structures enable interactions with the NP surface via hydrogen bonding and π–π stacking. This interaction forms a protective layer around the NPs, which not only prevents agglomeration but also contributes to controlling size and shape, thereby enhancing NP stability and functional performance.

Furthermore, the presence of these phytochemicals significantly influences the surface chemistry of the NPs, affecting their surface charge and interactions with the surrounding environment. This molecular-level stabilization not only extends the shelf life of the ZnONPs but also enhances their biocompatibility and functional performance, making them suitable for various applications, particularly in medicine and pharmacology. In addition to the bioactive compounds that contribute to the therapeutic properties of the studied plant species [29, 30, 33], the biosynthesized ZnONPs also exhibited notable paramagnetic properties, adding further value to their potential use in biomedical applications.

In the preclinical study, the bio-ZnONPs exhibited varying levels of interaction with the tested *in vitro* bacterial, yeast, and fungal strains. Antimicrobial activity was assessed based on the diameter of inhibition zones. A strong antimicrobial effect was defined by inhibition zones of 15 mm or greater, indicating high efficacy in suppressing microbial growth within a substantial radius from the disc. Moderate activity was associated with inhibition zones ranging from 10 to 14 mm, suggesting a noticeable but less potent antimicrobial effect. Weak activity was indicated by inhibition zones smaller than 10 mm, implying limited effectiveness or suboptimal NP concentration for achieving significant microbial inhibition.

*Thymhirs.bio*-ZnONP was the most effective against the bacterial strain *Staphylococcus aureus* ATCC 25923, the yeast *Candida albicans* ATCC 90028, and the fungal strains *Aspergillus flavus* and *Aspergillus fumigatus*. *Aloever*.bio-ZnONP exhibited the highest inhibitory activity against the bacterial strain *Micrococcus luteus* NCIMB 8166 and the yeasts *C. albicans*, *C. krusei*, and *C. neoformans*. It also demonstrated strong antifungal activity against all Aspergillus species tested, particularly *A. flavus* and *A. fumigatus*. *Retam*.bio-ZnONP showed notable antibacterial activity against *S. aureus*, *M. luteus*, and *Salmonella enterica* serotype Typhimurium, along with antifungal activity against *C. albicans* and *A. flavus*. *Harm*.bio-ZnONP was active against *S. aureus*, *M. luteus*, *C. neoformans*, and all tested *Aspergillus* species, with the strongest effect observed against *A. fumigatus.* Overall, these findings confirm the broad-spectrum antibacterial, antifungal, and antimicrobial potential of the biosynthesized ZnONPs.

The findings of this study align with previous research demonstrating the antimicrobial activity of ZnONPs against a range of pathogens, including *Escherichia coli*, *Bacillus subtilis*, *Salmonella spp*., *Listeria monocytogenes*, and *Staphylococcus aureus* [13, 15, 34]. Several mechanisms have been proposed to explain the antibacterial action of ZnONPs, including disruption of the microbial cell membrane and induction of intracellular damage through oxidative stress caused by the overproduction of reactive oxygen species (ROS). In a previous study, green-synthesized ZnONPs using *Passiflora caerulea* exhibited significant inhibition zones against pathogenic microbes, highlighting their potential in biomedical applications [34]. Similarly, biologically synthesized silver NPs (AgNPs) demonstrated strong antimicrobial activity without toxic effects on human or mouse cells, suggesting a promising capacity to selectively target pathogens while sparing mammalian cells [4].

The findings of this study highlight the potential of the selected plant species as effective and sustainable tools for the biosynthesis of antimicrobial bio-ZnONPs, with promising applications across multiple domains. The differential antimicrobial responses observed among Thymhirs.bio-ZnONP, Aloever.bio-ZnONP, Retam.bio-ZnONP, and Harm.bio-ZnONP against various microorganisms suggest their suitability for development as targeted bio-nanoproducts. Integrating plant phytochemistry with appropriate processing and nanosynthesis techniques may enhance the therapeutic or industrial efficacy of the final nanomaterials. Plant-based ZnONPs offer a unique advantage by improving the functional potential of bioactive plant compounds, particularly in medicinal and sustainable industrial applications. For instance, T. hirsuta-derived ZnONPs may hold promise for improved clinical outcomes, while A. vera-based ZnONPs could be valuable in the pharmaceutical and food industries due to their bioactivity and safety profile [35, 36]. R. monosperma-derived ZnONPs may contribute to skin repair and wound healing [37]. Additionally, R. monosperma is a potential source of natural fibers for the textile industry, offering benefits, such as abundance, easy extraction, biodegradability, non-toxicity, eco-friendliness, and recyclability. The combination of these properties with the antimicrobial effects of ZnONPs could further enhance the value of these fibers for industrial applications [38]. Furthermore, P. harmala has well-documented pharmacological potential, including antimicrobial, anticancer, and multi-organ protective effects involving the cardiovascular, nervous, endocrine, GI, and respiratory systems. Its richness in phenolic compounds also confers notable antioxidant properties, supporting its use in therapeutic formulations. The combination of such high-potential medicinal plants with the physicochemical and biological features of ZnONPs may synergistically enhance the efficacy and safety of future nanomedicine formulations.

Nonetheless, further research is required to develop optimized processing techniques that can preserve—or even enhance—the bioactive properties naturally present in plants. Such advancements would contribute to improved product quality, safety, and functional performance. Additionally, industries involved in processing plant-based materials for food and medicinal applications must rigorously assess the safety and toxicological profiles of bio-engineered nanomaterials to ensure their suitability for human use.

The lipophilicity, drug-likeness, and pharmacokinetic properties of the phenolic acids and flavonoids identified in *Thymelaea hirsuta L*., *Aloe vera L*., *Retama monosperma L*., and *Peganum harmala L*. are summarized in Table 5. Assessing these parameters is critical in drug design and development to help prevent failures during advanced stages of drug discovery [26, 27, 29, 30, 33]. Most of the studied phytochemicals comply with Lipinski’s Rule of Five, indicating favorable druggability. Key physicochemical properties—including flexibility (Flex), unsaturation (Insatu), insolubility (Insolu), lipophilicity (Lipo), molecular size (Size), and polarity (Pola)—revealed acceptable oral bio-availability scores ranging from 0.11 to 0.85, supporting the compounds’ potential bioactivity. Both BBB permeability and GI absorption were evaluated and visualized using the BOILED-Egg model (Figure 5). Most of the identified compounds also showed acceptable skin permeability, with Log Kp values ranging from −5.66 to −9.15, and were not predicted to be substrates of P-gp. Given the crucial role of cytochrome P450 enzymes—such as CYP1A2, CYP2C9, CYP2C19, CYP2D6, and CYP3A4—in drug metabolism and excretion [29, 30, 39], potential inhibition of these enzymes was also assessed. Table 4 shows that while some phytochemicals inhibited individual CYP isoforms, none inhibited CYP2C19. These findings suggest a favorable safety profile concerning metabolic interactions. Lastly, the synthetic accessibility scores for these compounds ranged from 1.07 to 5.28, indicating that they are generally easy to synthesize and thus viable candidates for further drug development [29, 30, 39].

All of the analyzed phytochemicals were predicted to exhibit negative, yet varied, binding affinities toward the three target receptors: 1JIJ (TyrRS), 2QZW (secreted aspartic proteinase), and 2UVO (wheat germ agglutinin) (Table 5). The highest binding affinities were −10.5, −10.0, and −8.4 kcal/mol for 1JIJ, 2QZW, and 2UVO, respectively. These variations in binding strength are attributed to differences in the three-dimensional chemical structures of both ligands and receptors [26, 29, 33]. The best binding scores corresponded to phytochemicals Nos. 12, 8, and 16, respectively (Tables 5 and 6). These compounds are known to possess multiple biological activities, including potent antimicrobial effects [30, 40], promotion of apoptosis, and inhibition of cancer cell lines, such as HeLa and MCF-7 [30, 41]. Molecular docking revealed that several phytochemicals formed stable interactions with the target receptors, including up to nine conventional hydrogen bonds. For example, compound No. 8 established nine H-bonds, supported by van der Waals and π-alkyl interactions, which contribute to the stability of the ligand-receptor complex (Figure 6) [26, 27, 29]. The key interacting residues for compound No. 8 included LYS49, SER89, ASN160, LYS178, SER336, GLY87, PRO4, ILE338, and THR166. Notably, THR166 formed three hydrogen bonds with compound No. 8 and was the closest interacting residue, at a distance of 2.150 Å. Such deep embedding (<2.5 Å) of ligands within the receptor binding pocket has been associated with significant biological effects, including anticancer, anti-inflammatory, and antimicrobial properties [39, 42].

Deep embedding of ligands at distances less than 2.5 Å, as observed in this study, has been widely reported to contribute to various biological activities, including antioxidant, anti-proliferative, anti-inflammatory, and antimicrobial effects [24, 39, 43]. Based on the strong binding affinities, extensive molecular interactions, and deep embedding of the identified phytochemicals, it can be inferred that their antimicrobial effects are thermodynamically feasible. These predictions are consistent with our *in vitro* findings, supporting the potential health-promoting properties and phytotherapeutic value of medicinal plants [24, 39, 42]. Despite these promising outcomes, several limitations currently hinder the large-scale application and reproducibility of plant-mediated ZnONPs. One major challenge lies in the variability of synthesis, which is heavily influenced by the biochemical composition of the plant extract—dependent on plant species, environmental conditions, and extraction methods. This variability often leads to inconsistencies in NP properties, such as size, morphology, and stability. Compared to chemical or physical methods, plant-based synthesis offers limited control over these parameters, often resulting in broader particle size distributions and irregular shapes [44]. Another limitation involves the lower long-term stability of plant-derived ZnONPs due to weaker capping by organic compounds, which can diminish their effectiveness over time [45]. Additionally, the complex mixture of phytochemicals in plant extracts can introduce impurities, necessitating further purification steps to ensure quality and functionality. Industrial-scale production also poses challenges due to the difficulty of maintaining consistent precursor concentrations, reaction times, and temperatures when using plant-based systems. Furthermore, the precise mechanisms by which plant metabolites reduce and stabilize ZnONPs remain poorly understood, limiting the optimization of synthesis protocols for specific biomedical applications. To overcome these challenges, further research is required to assess NP stability under various physiological conditions, including pH changes, enzymatic activity, and the presence of proteins in biological fluids. We recommend developing optimization strategies that include fine-tuning key reaction parameters—such as temperature, pH, reaction time, and precursor concentration—to achieve consistent particle size, shape, and surface characteristics. Employing continuous flow reactors may enhance scalability and reproducibility by allowing for precise control of reaction conditions. Additionally, microfluidic platforms offer potential for uniform NP synthesis at the microscale, reducing batch-to-batch variation. Robust characterization techniques should be implemented at multiple stages of synthesis to monitor NP attributes and ensure product consistency. Moreover, the integration of automated systems for NP synthesis and purification could streamline production, minimize human error, and improve scalability. By adopting these strategies, it is possible to scale up the production of plant-derived ZnONPs while preserving their structural integrity and antimicrobial efficacy. This would significantly advance their practical applications in biomedical and pharmaceutical fields, making plant-based nanomaterials a viable alternative to conventional synthesis approaches.

Addressing safety protocols and environmental impacts is essential when scaling up the production of plant-derived ZnONPs. This includes implementing proper waste management strategies and ensuring the safe disposal of reactants and by-products generated during synthesis. Collaboration with industry partners, research institutions, and regulatory agencies is also recommended to validate the scalability, safety, and efficacy of large-scale NP production processes. Although plant-based ZnONPs are often regarded as biocompatible, further research is necessary to fully assess their potential toxicity and suitability for biomedical applications. Comprehensive evaluations of cytotoxicity, immunogenicity, and long-term effects are critical to ensure their safe integration into clinical settings. Special attention should be given to how these NP interact with biological systems, including their potential to induce oxidative stress, inflammatory responses, or unintended cellular damage. For example, ZnONPs synthesized from *Eclipta prostrata* have been shown to exhibit cytotoxic effects on human lung fibroblast cells at higher concentrations [46], highlighting the need for careful dose optimization and safety profiling. These findings emphasize that, despite their natural origin, plant-derived nanomaterials must undergo rigorous biocompatibility assessments before their use in both biomedical and environmental applications.

## Conclusion

Overall, this study demonstrated a rapid, cost-effective, and environmentally friendly method for the biosynthesis of ZnONPs using under-utilized plant species native to temperate desert and Mediterranean regions—*Thymelaea hirsuta L*., *Aloe vera L*., *Retama monosperma L*., and *Peganum harmala L*. These biologically synthesized NPs represent promising nanoproducts with potential applications in developing more effective antimicrobial agents, particularly against drug-resistant pathogens, as well as in functional foods and pharmaceutical formulations. The observed *in vitro* antimicrobial activities can be partially attributed to the pharmacokinetic properties and molecular interactions of phytochemicals involved in ZnONP biosynthesis. These interactions, particularly with known antimicrobial target receptors, may enhance the biological efficacy of the resulting NPs, providing a foundation for future therapeutic applications.

## Supplemental data

Supplementary data are available at the following link: https://www.bjbms.org/ojs/index.php/bjbms/article/view/12090/3883.

## Data Availability

The data supporting the findings of this study are available upon request.
